# Inhibition of PI3K by copanlisib exerts potent antitumor effects on Merkel cell carcinoma cell lines and mouse xenografts

**DOI:** 10.1038/s41598-020-65637-2

**Published:** 2020-06-01

**Authors:** Bin Fang, Aarthi Kannan, Stephanie Zhao, Quy H. Nguyen, Samuel Ejadi, Maki Yamamoto, J. Camilo Barreto, Haibo Zhao, Ling Gao

**Affiliations:** 1 Department of Dermatology, College of Medicine, University of Arkansas for Medical Sciences, Little Rock, AR USA; 2 Department of Nephrology, Union Hospital, Tongji Medical College, Huazhong University of Science and Technology, Wuhan, Hubei China; 3 Southern California Institute of Research and Education, Long Beach, CA USA; 4 Department of Dermatology, University of California – Irvine, Irvine, CA USA; 5 Harvard College, Cambridge, MA USA; 6 Department of Biological Chemistry, University of California – Irvine, Irvine, CA USA; 7 Division of Hematology/Oncology, School of Medicine, University of California – Irvine, Irvine, CA USA; 8 Department of Surgery, School of Medicine, University of California – Irvine, Irvine, CA USA; 9 Department of Surgery, College of Medicine, University of Arkansas for Medical Sciences, Little Rock, AR USA; 10 Veterans Affairs Long Beach Healthcare System, Long Beach, CA USA

**Keywords:** Head and neck cancer, Skin cancer, Cancer, Targeted therapies

## Abstract

Merkel cell carcinoma (MCC) is a highly aggressive neuroendocrine skin cancer with steadily increasing incidence and poor prognosis. Despite recent success with immunotherapy, 50% of patients still succumb to their diseases. To date, there is no Food and Drug Administration-approved targeted therapy for advanced MCC. Aberrant activation of phosphatidylinositide-3-kinase (PI3K)/AKT/mTOR pathway is frequently detected in MCC, making it an attractive therapeutic target. We previously found PI3K pathway activation in human MCC cell lines and tumors and demonstrated complete clinical response in a Stage IV MCC patient treated with PI3K inhibitor idelalisib. Here, we found that both PI3K-α and -δ isoforms are abundantly expressed in our MCC cell lines and clinical samples; we therefore examined antitumor efficacy across a panel of five PI3K inhibitors with distinctive isoform-specificities, including idelalisib (PI3K-δ), copanlisib (PI3K-α/δ), duvelisib (PI3K-γ/δ), alpelisib (PI3K-α), and AZD8186 (PI3K-β/δ). Of these, copanlisib exerts the most potent antitumor effects, markedly inhibiting cell proliferation, survival, and tumor growth by suppressing PI3K/mTOR/Akt activities in mouse models generated from MCC cell xenografts and patient-derived tumor xenografts. These results provide compelling preclinical evidence for application of copanlisib in advanced MCC with aberrant PI3K activation for which immunotherapy is insufficient, or patients who are unsuitable for immunotherapy.

## Introduction

Merkel cell carcinoma (MCC) is a highly aggressive neuroendocrine malignancy of the skin with steadily increasing incidence^1–3^. Chronic ultraviolet-light (UV) exposure^4^, clonal integration of Merkel cell polyomavirus (MCPyV)^5^, immunosuppression^6^, and aging^2^ are common risk factors for MCC. MCC is an often lethal tumor with high recurrence, and the overall 5-year survival rate is 0–18% for advanced MCC^7,8^, making it deadlier than melanoma^9,10^. Nevertheless, cellular origin and molecular events driving MCC tumorigenesis remain unknown.

Recent immunotherapy targeting the PD1/PD-L1 (programmed cell death protein 1/PD1 ligand) immune checkpoint pathway has demonstrated durable response rates and clinical benefits^11–13^, indicating that tumoral immune cell infiltration and function play an important role in MCC development, growth, and clinical outcomes^14–16^. Both pembrolizumab (humanized anti-PD-1 antibody) and avelumab (humanized anti-PD-L1 antibody) have been approved by the Food and Drug Administration (FDA) for treatment of advanced MCC^17,18^. However, a significant portion (~50%) of MCC patients either fail to respond to immune checkpoint inhibitors or develop acquired resistance. To tackle these problems, combinations of different immunotherapies for treatment of metastatic MCCs are being evaluated in clinical trials^17,18^. Meanwhile, there is an emerging paradigm in cancer therapy to combine immunotherapy with molecularly targeted therapies that, in addition to their direct cell-autonomous effects on tumor cells, may boost therapeutic response rate and efficacy of immunotherapies^19–21^. These targeted therapies can also be used to treat MCC patients who are not suitable for immunotherapy due to a variety of autoimmune diseases or immunosuppressive conditions such as HIV infection and organ transplantation. However, to date, there is no FDA-approved molecularly targeted therapy for treatment of MCC.

Genomic studies of MCCs, especially MCPyV-negative tumors, have identified chromosomal copy number variations (CNVs) and frequent mutations in tumor suppressor genes such as *TP53* and *RB1* (retinoblastoma 1)^22,23^, several oncogenes including *HRAS*, *KRAS*^24,25^, and genes encoding phosphatidylinositide-3-kinase (PI3K)/AKT/mTOR pathway^26–28^. Unlike other solid tumors, activating mutations in receptor tyrosine kinases of growth factors are not detected in MCCs and the mitogen-activated protein kinase (MAPK) signaling pathway is not constitutively activated in MCCs^29^. PI3K/AKT/mTOR pathway regulates many cellular processes that are involved in carcinogenesis including cell cycle/proliferation, differentiation, survival, motility, and metabolism^30–32^. This pathway is one of the most overactive pathways in a broad spectrum of solid tumors and hematological malignancies, making PI3K pathway an attractive therapeutic target for cancer treatment. Class I PI3Ks are activated by receptor tyrosine kinases of insulin, growth factor receptors, and G protein-coupled receptors of hormones and chemokines. They are heterodimers composed of a regulatory subunit (p85α, p55α, p50α, p85β, and p55γ encoded by *PIK3R1, PIK3R2, and PIK3R3*, respectively) and a catalytic subunit (PI3K-α, PI3K-β, PI3K-δ, and PI3K-γ encoded by *PIK3CA, PIK3CB, PIK3CD*, and *PIK3CG*, respectively). PI3Ks trigger the generation of an important lipid second messenger phosphatidylinositol (3,4,5)-trisphosphate (PI(3,4,5)P3), which then recruits and activates multiple downstream signaling pathways including AKT and mTOR^33^.

Numerous pan- and isoform-specific PI3K inhibitors have been developed and are being tested at different stages of clinical trials^34–36^. Four PI3K inhibitors have been FDA-approved so far for treatment of various leukemias and solid tumors. Idelalisib (CAL-101), a highly specific PI3K-δ inhibitor, was approved for treatment of relapsed chronic lymphocytic leukemia (CLL), relapsed follicular B-cell non-Hodgkin lymphoma (NHL) and relapsed small lymphocytic leukemia (SLL)^37^. Copanlisib (BAY 80–6946), a reversible pan-class PI3K inhibitor with predominant activity against PI3K-α and -δ isoforms, was approved for refractory follicular lymphoma (FL)^38^. Duvelisib (IPI-145), a dual inhibitor of PI3K-δ and -γ, was approved for treatment of adult patients with relapsed or refractory CLL, SLL, and FL after at least two prior systemic therapies^39^. More recently, alpelisib (BYL719), an oral PI3Kα-specific inhibitor, was approved for treatment of *PIK3CA*-mutated hormone receptor-positive advanced breast cancer^40^.

Our group and others have detected activating mutations of *PIK3CA* and frequent activation of PI3K/AKT/mTOR pathway in MCC tumors, thus indicating PI3Ks and downstream signaling molecules are good therapeutic targets. Pan-PI3K inhibitors remarkably suppress MCC growth and survival^26–28,41^; however, pan-PI3K inhibitors have limited clinical application due to severe side effects^42–46^. Thus, recent drug development has focused on PI3K isoform-specific inhibitors^31,46^. We reported the case of a stage IV MCC patient with *PIK3CA* mutation who demonstrated a complete clinical response to idelalisib^47^. This was the first successful application of a PI3K inhibitor in advanced MCC and of a PI3K-δ inhibitor in a solid tumor. Moreover, this was the first report of PI3K-δ isoform expression in primary human MCC cells, which has since been independently confirmed by another study^48^. Additionally, we have demonstrated that MLN0128, a second generation dual TORC1/2 inhibitor, significantly attenuated MCC tumor growth in MCC cell line-derived (CDX) mouse models^49^, thus confirming that this pathway is a valid therapeutic target in MCC.

Although traditional animal models of human cancers utilizing CDX remain a classic and powerful tool to evaluate drug efficacy and toxicity, these models are not wholly representative of primary tumor heterogeneity. Thus, CDX models provide initial preclinical evidence but may lack predictive power for how patients will respond in the clinical setting^50,51^. By preserving primary tumor characteristics and heterogeneity, patient-derived tumor xenograft (PDX) models provide an advantage over classical CDX models, and recent studies have demonstrated that PDX models of cancer have great value in predicting actual clinical response to anticancer agents^52–57^. Towards this end, we recently established and characterized multiple PDX lineages of MCC. Therefore, for the first time in MCC studies, we have been able to validate drug efficacy using PDX models of MCC.

In the present study, in addition to confirming high PI3K-δ expression in 52% of MCC tissues, we found elevated PI3K-α expression in 70% of archival MCC tumor samples. Given the differential expression of PI3K isoforms in MCC, we examined antitumor efficacy of four different FDA-approved PI3K isoform-specific inhibitors (idelalisib, copanlisib, duvelisib, and alpelisib) as well as AZD8186, a dual PI3K-β/δ inhibitor currently in advanced clinical development. Copanlisib exerted the most potent anti-tumor growth effects on MCC cells by suppressing PI3K/mTOR/Akt activities. Furthermore, copanlisib markedly repressed *in vivo* tumor growth in MCC mouse models generated from MCC cells and patient tumors. Together, these findings provide a compelling rationale for copanlisib as a monotherapy or potentially as part of a combinatorial therapeutic regimen for advanced MCC.

## Results

### Expression of PI3K- isoforms of class I PI3K catalytic subunit in MCC cell lines and tumors

**Figure 1:**
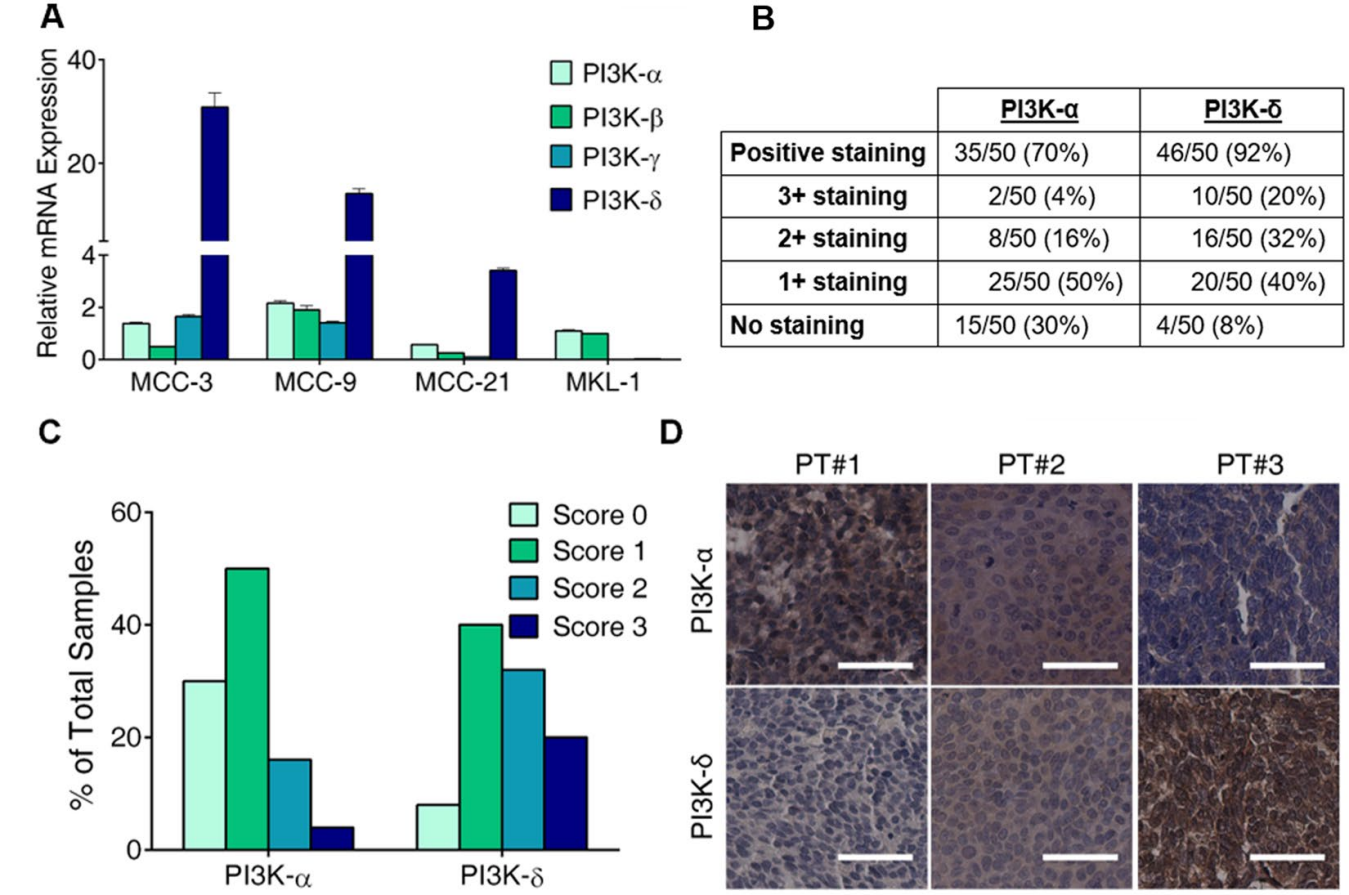
Expression of PI3K- isoforms of class I PI3K in MCC cell lines and tumors. (**A**) Relative mRNA expression of PI3K- isoforms in four MCC cell lines detected by qPCR. mRNA expression of target genes was normalized to that of *MRPS2* (mitochondrial ribosomal protein S2). Data from triplicate runs presented as mean ± SD. (**B**) Expression of PI3K-α and PI3K-δ in 50 archived human MCC tumor samples detected by immunohistochemistry. Staining intensity graded as 0, 1+ , 2+ and 3+ for negative, low, moderate, and high expression, respectively. (**C**) Distribution of PI3K-α and PI3K-δ expression in human MCC samples. (**D**) Representative immunohistochemistry staining of PI3K-α and PI3K-δ in human Merkel cell carcinomas. scale bar = 50 μm.

We and others have previously demonstrated that the PI3K/mTOR/Akt pathway is commonly activated in MCC tumors^27,28,49,58^. To quantify the mRNA expression of class I PI3K catalytic subunit isoforms (PI3K-α, PI3K-β, PI3K-δ, and PI3K-γ) in MCC cell lines, real time quantitative RT-PCR (qPCR) was conducted using cDNAs isolated from three primary MCC cell lines (MCC-3, MCC-9, and MCC-21) established in our laboratory as well as MKL-1, a commercially available classic MCC cell line. Among these cell lines, MCC-3 and MCC-9 are MCPyV-negative, while MCC-21 and MKL-1 are MCPyV-positive. As shown in Fig. 1A, mRNA expression of all four isoforms were detected in MCC-3, −9, and −21 with PI3K-δ being the most abundantly expressed. Only PI3K-α and -β were expressed in MKL-1. Next, we set out to examine PI3K-α and -δ expression in 50 primary MCC archived tissue samples by immunohistochemistry with isoform-specific antibodies. Histologic grading, ranging from negative (score 0) to high expression (score 3), demonstrated that 20% (10 of 50 MCC tumors) had high expression (score 2 and score 3) of PI3K-α isoform, whereas 30% (15 of 50) had no detectable expression (score 0). High PI3K-δ expression was observed in 52% (26 out of 50) of MCC tumors, and no PI3K-δ was detected in 8% of samples (Fig. 1B,C). Representative immunohistochemistry staining of PI3K-α and -δ in human MCC samples are shown in Fig. 1D. These results demonstrate that class I PI3K isoforms are differentially expressed in MCC cell lines and tissue samples, and indicate that MCCs may respond distinctively to isoform-specific PI3K inhibitors.

### Inhibition of PI3K- α/δ by copanlisib elicits the most potent antitumor effects on MCC cell lines compared to other PI3K isoform-selective inhibitors

**Figure 2:**
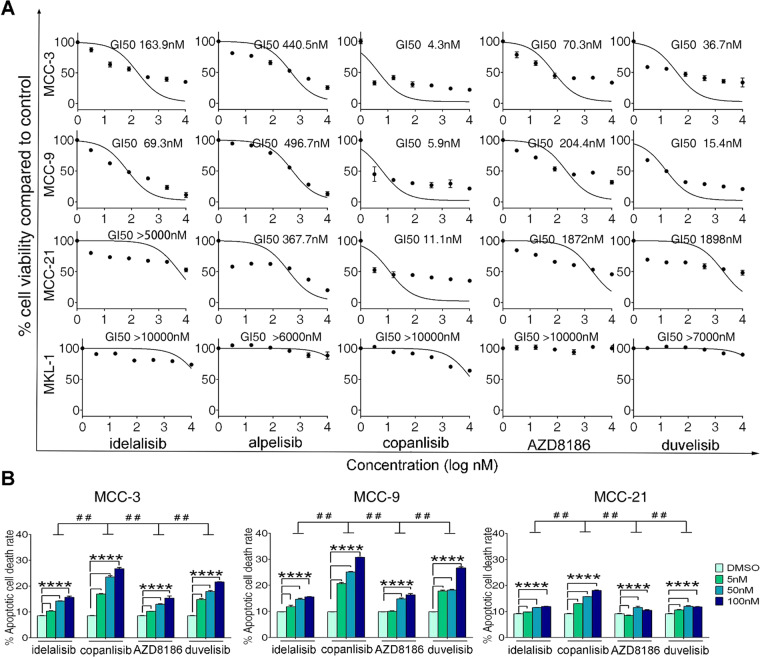
PI3K inhibitors suppress cell growth and induce apoptosis in MCC *in vitro*. (**A**) Cultured cells of four MCC cell lines were treated with serial concentrations of idelalisib, alpelisib, copanlisib, AZD8186 and IPI145 duvelisib for 72 hours and cell viability was assessed by CCK-8 assay. Maximal cell viability (100%) was defined as average viability of DMSO-treated samples and half maximal growth inhibitory concentration was calculated. Data are presented as mean ± SD from triplicate experiments. (**B**) MCC-3, MCC-9, and MCC-21 cells were treated with 5 nM, 50 nM and 100 nM of idelalisib, copanlisib, AZD8186, and duvelisib for 24 hours, respectively. DMSO-treated cells served as negative controls. Cells were stained by Annexin-V and PI (propidium iodide) and analyzed by flow cytometry; percentages of Annexin V^+^, PI^−^ (early apoptotic) and Annexin-V^+^, PI^+^ (late apoptotic) cells were calculated in each group. Bar graphs represent all dead cells including Annexin V^+^, PI^−^ cells and Annexin-V^+^, PI^+^ cells. Data are presented as mean ± SD from quadruplicate experiments and *n* = 3. ****p < 0.0001 versus DMSO-treated cells; ^##^*p* < 0.01 versus idelalisib-, AZD8168-, and duvelisib-treated cells by one-way ANOVA.

Next, we tested the responses of the above four MCC cell lines to different PI3K inhibitors, which have distinctive isoform-selectivity, including idelalisib, alpelisib, copanlisib, AZD8186, and duvelisib. The antitumor efficacy of these inhibitors at a series of concentrations from 0 to 10 µM on MCC cell lines was measured by CCK-8 assay, which has been used for assessment of cell viability and proliferation. The half-maximal growth inhibitory concentration (GI_50_) of these inhibitors on different MCC cell lines was calculated as described previously^49^ and shown in Fig. 2A. MCC-3 and MCC-9 cell viability and proliferation was suppressed by all five PI3K inhibitors. Among them, dual-isoform specific inhibitors (copanlisib, AZD8186, and duvelisib) generally showed more potency than single-isoform inhibitors (alpelisib and idelalisib), though MCC-9 was more sensitive to idelalisib (PI3K-δ) than AZD8186 (PI3K-β/δ). Interestingly, inhibition of PI3K-δ (idelalisib) exerted more potent anti-tumor growth effect than PI3K-α inhibition (alpelisib) in MCC-3 and MCC-9 cells, which display predominant PI3K-δ mRNA expression (Fig. 1A). However, this was not the case in MCC-21 cells; although PI3K-δ is highly expressed in MCC-21, this cell line responded poorly to idelalisib (PI3K-δ), AZD8186 (PI3K-β/δ), and duvelisib (PI3K-γ/δ). Instead, MCC-21 proliferation was well repressed by inhibition of PI3K-α (alpelisib) and PI3K-α/δ (copanlisib), suggesting that predominant isoform expression does not fully correlate to responsiveness. Although the underlying mechanisms are potentially intriguing and require further investigation, copanlisib (PI3K-α/δ) demonstrated the most potent anti-tumor efficacy on MCC-3, MCC-9, and MCC-21 cell lines, in which PI3K-δ and -α are the two most abundantly expressed PI3K isoforms. In contrast, MKL-1 cells, which we found had negligible expression of PI3K isoforms, were resistant to all PI3K inhibitors tested (Fig. 2A). Finally, since inhibition of PI3K-α alone by alpelisib showed less anti-tumor potency on MCC-3 and MCC-9 than other inhibitors, alpelisib was excluded from further experiments.

We then further examined the effects of PI3K inhibitors on apoptosis of MCC-3, MCC-9, and MCC-21 cell lines. Cultured MCC cells were treated with three doses (5 nM, 50 nM and 100 nM) of idelalisib, copanlisib, AZD8186 and duvelisib for 24 hours, respectively. DMSO treatment served as a respective negative control for each cell line. Apoptotic rate was measured by Annexin-V and PI (propidium iodide) staining followed by flow-cytometry analysis (Fig. 2B). All four inhibitors induced apoptosis in all three MCC cell lines in a dose-dependent manner with the more prominent effect observed in MCC-3 and MCC-9. Consistently, inhibition of PI3Kα/δ by copanlisib at three doses resulted in the most robust anti-MCC survival effect on three MCC cell lines (Fig. 2A). Though MKL-1 failed to respond to all PI3K inhibitors tested, we wanted to examine if a higher dose of copanlisib induced apoptosis. MKL-1 cells were treated with copanlisib 1 µM for 24 h, followed by Annexin-V and PI staining and flow cytometry analysis; copanlisib exerted negligible apoptotic effect on MKL-1 cells (Supplementary Fig. 4). In summary, these data indicate that inhibition of PI3K α/δ isoforms by copanlisib had the most potent antitumor growth and survival effects on MCC compared to other PI3K inhibitors.

### Copanlisib suppresses MCC colony formation by inhibiting MCC cell proliferation and survival *in vitro*

**Figure 3:**
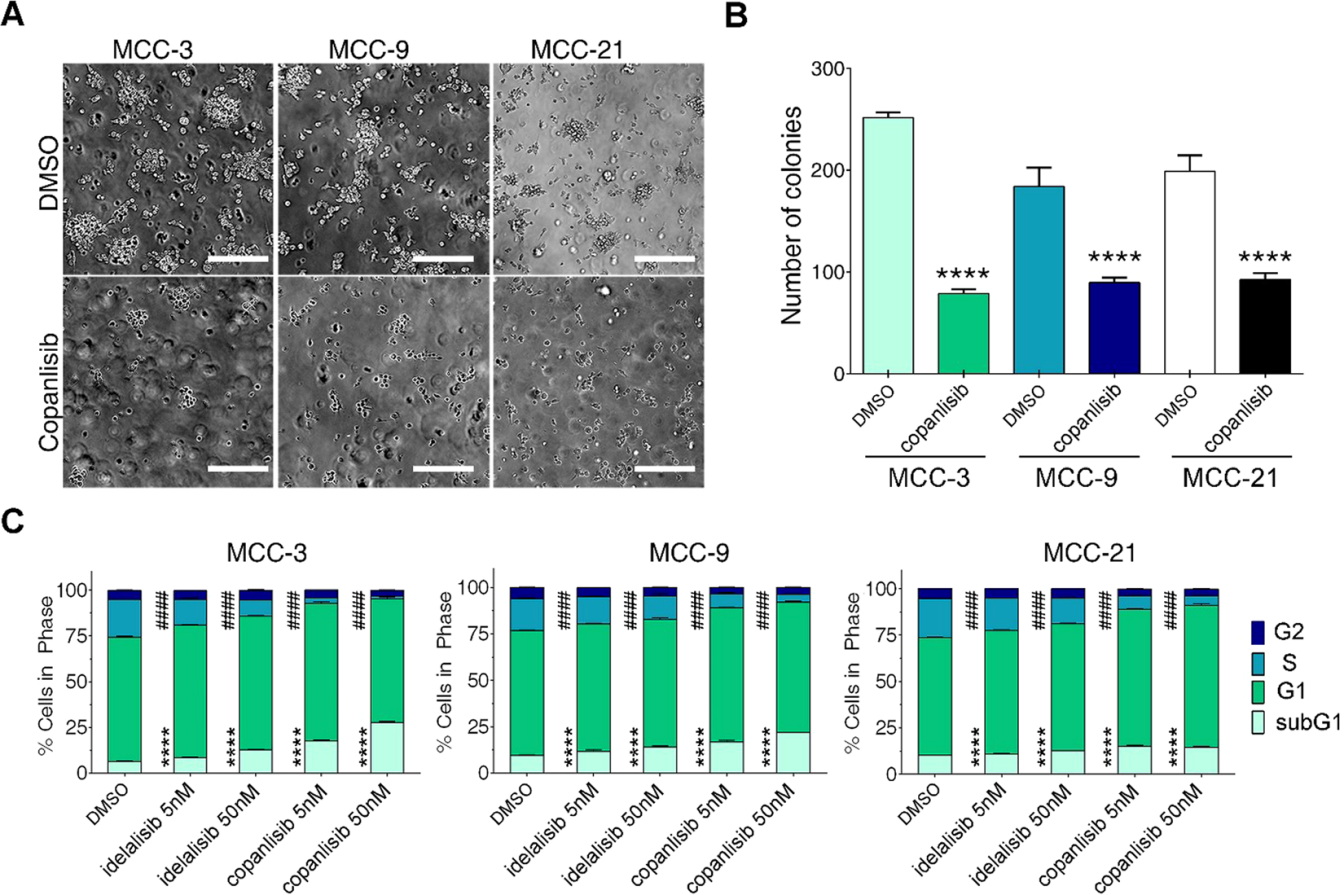
Copanlisib suppresses MCC clonogenic formation by inhibiting cell proliferation and inducing apoptosis in MCC cells. (**A**) Decreased colony formation in MCC cells treated with copanlisib. MCC-3, MCC-9, and MCC-21 cells were plated in methylcellulose medium with DMSO or copanlisib (50 nM) and cultured for 14 days at 37 °C; colonies were assessed on Day 14. Representative images were taken at 40x magnification from different MCC culture plates. (**B**) Number of colonies in each plate. Data are presented as mean ± SD from triplicate experiments. ****p < 0.0001 compared with DMSO-treated cells by two-sided *Student t-test* and *n* = 4. (**C**) MCC-3, MCC-9, and MCC-21 cells were treated with 5 nM and 50 nM of idelalisib or copanlisib for 24 hours. Cells were stained by BrdU and 7-AAD and the cell cycle progression was analyzed by flow cytometry. Histograms show percentage of MCC cell population in sub-G1, G1, S, and G2 cell cycle phases. Data are presented as mean ± SD of triplicate experiments and *n* = 3. ^####^p < 0.0001, ****p < 0.0001 versus DMSO-treated cells by one-way ANOVA.

To assess the effect of copanlisib on MCC tumorigenesis *in vitro*, we performed a clonogenic assay on three MCC cell lines (MCC-3, MCC-9, and MCC-21) responsive to copanlisib treatment in the previous experiment (Fig. 2). As shown in Fig. 3A,B, copanlisib treatment significantly decreased the number of colonies formed in methylcellulose medium compared to that in vehicle-treated MCC cells. To further identify the mechanisms by which copanlisib suppresses MCC colony formation, we analyzed cell-cycle progression by flow cytometry in vehicle and copanlisib-treated MCC cell lines. For this purpose, MCC-3, MCC-9, and MCC-21 cells were treated with vehicle or idelalisib or copanlisib at 5 nM and 50 nM concentrations for 24 hours, respectively. Cells were then collected and subjected to BrdU (Bromodeoxyuridine) and PI (propidium iodide) fluorescent staining followed by flow cytometry analysis. Both idelalisib and copanlisib significantly decreased cell populations at S phase, an index of cell proliferation, compared to vehicle-treated controls. Meanwhile, the percentage of apoptotic cells, represented by sub-G1 cell population with DNA fragmentation, significantly increased in idelalisib- and copanlisib-treated MCC cells relative to controls (Fig. 3C). Consistent with the results shown in Fig. 2A, copanlisib exhibited stronger anti-tumor effects than idelalisib. These data indicate that copanlisib attenuates MCC growth *in vitro* by inhibiting MCC cell proliferation and inducing apoptosis.

### Copanlisib is more potent than idelalisib in suppressing PI3K/AKT/mTOR pathway in MCC cells

**Figure 4:**
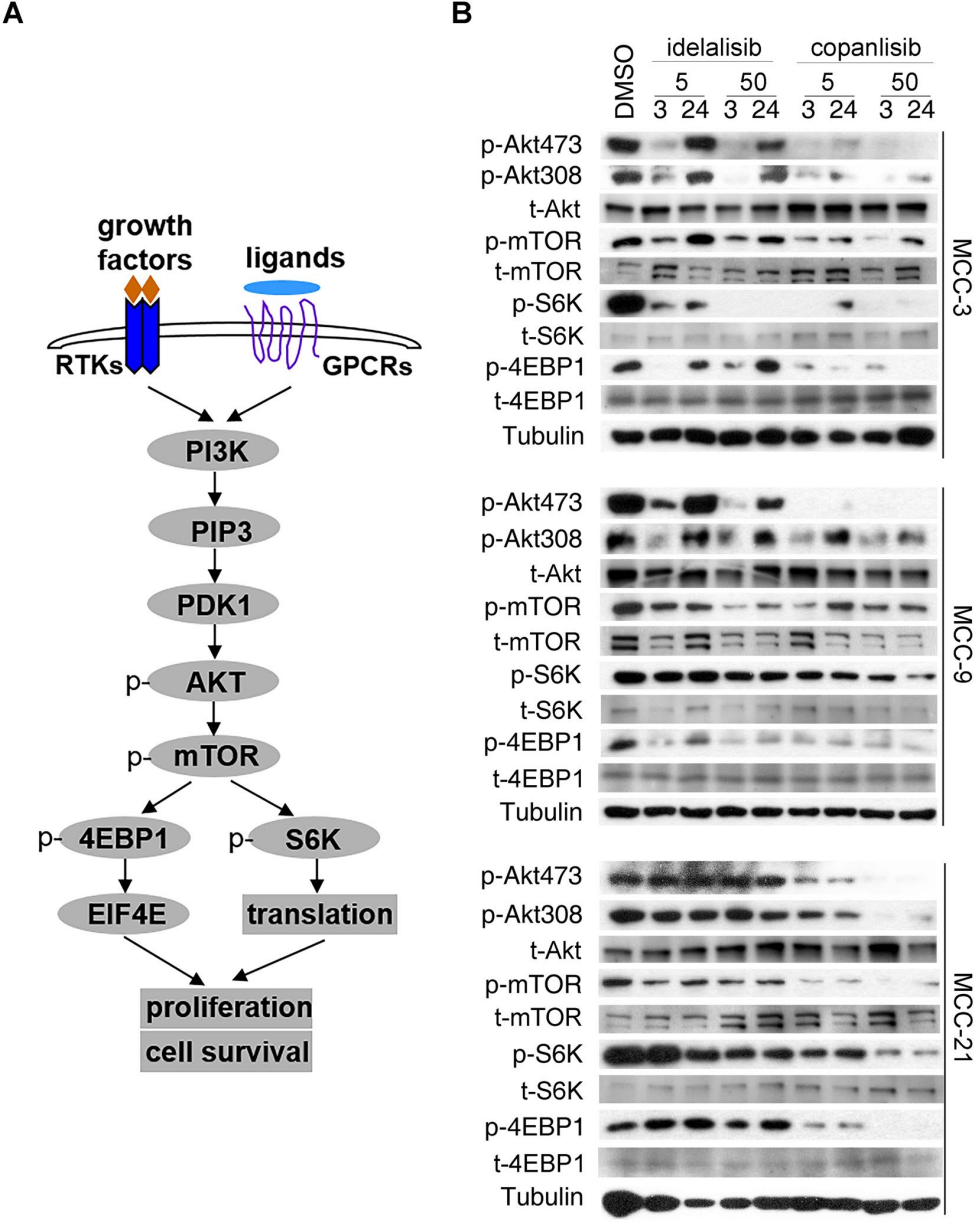
Copanlisib is more potent than idelalisib in inhibiting PI3K/AKT/mTOR pathway in MCC. (**A**) A cartoon illustration of activation of PI3K/AKT/mTOR pathway and downstream targets. RTKs, receptor tyrosine kinases; GPCRs, G-protein coupled receptors. (**B**) Cultured MCC-3, MCC-9, and MCC-21 cells were incubated with DMSO or 5 nM/50 nM of idelalisib and copanlisib, respectively, for 3 and 24 hours. Whole cell protein lysates (10–30 µg per lane) from the same experiment were prepared in parallel and resolved by SDS-PAGE gel electrophoresis, and subjected to immunoblotting with specific antibodies against phosphorylation of Akt at serine 473 and threonine 308, mTOR, and its downstream targets, S6K and 4EBP1, and respective total proteins. Blots were cropped from different parts of the same gels and analyzed by radiography with similar exposure conditions. All data represent contiguous lanes, and representative blots from triplicate experiments are shown here.

We next set out to examine the efficacy of idelalisib and copanlisib in decreasing activities of PI3K and its downstream AKT and mTOR pathways (Fig. 4A). Cells from three MCC cell lines (MCC-3, −9, −21) were treated with vehicle or 5 nM/50 nM of idelalisib and copanlisib for 3 and 24 hours, respectively. Vehicle-treated cells served as negative controls. Whole cell protein lysates were prepared and PI3K pathway activation, as revealed by phosphorylation of AKT, mTOR and their downstream targets, was detected by western blots using specific antibodies as described in Materials and Methods (Fig. 4B). Consistent with the GI_50_ data as shown in Fig. 2A, copanlisib inhibited phosphorylation and activation of AKT/mTOR pathway more robustly than idelalisib in all three MCC cell lines (Fig. 4B). Reflecting the inability of idelalisib to suppress MCC-21 cell proliferation (Fig. 2A), we found that idelalisib had little effect on AKT and mTOR activation in this cell line at both 5 nM and 50 nM concentrations after treatment for 3 and 24 hours (lower panel in Fig. 4B). In contrast, both idelalisib and copanlisib induced quick reduction in phosphorylation of PI3K downstream signaling molecules in MCC-3 and MCC-9 after 3-hour incubation. We observed a rebound of AKT and mTOR phosphorylation after a 24-hour incubation with these PI3K inhibitors, which was more apparent in idelalisib-treated MCC-3 and MCC-9 (upper and middle panels in Fig. 4B). These data demonstrate that inhibition of both PI3K-α and -δ isoforms by copanlisib represses PI3K/AKT/mTOR pathway in MCC cells more potently than idelalisib.

### Copanlisib attenuates MCC xenograft tumor growth *in vivo* by inhibiting MCC proliferation and stimulating apoptosis

**Figure 5:**
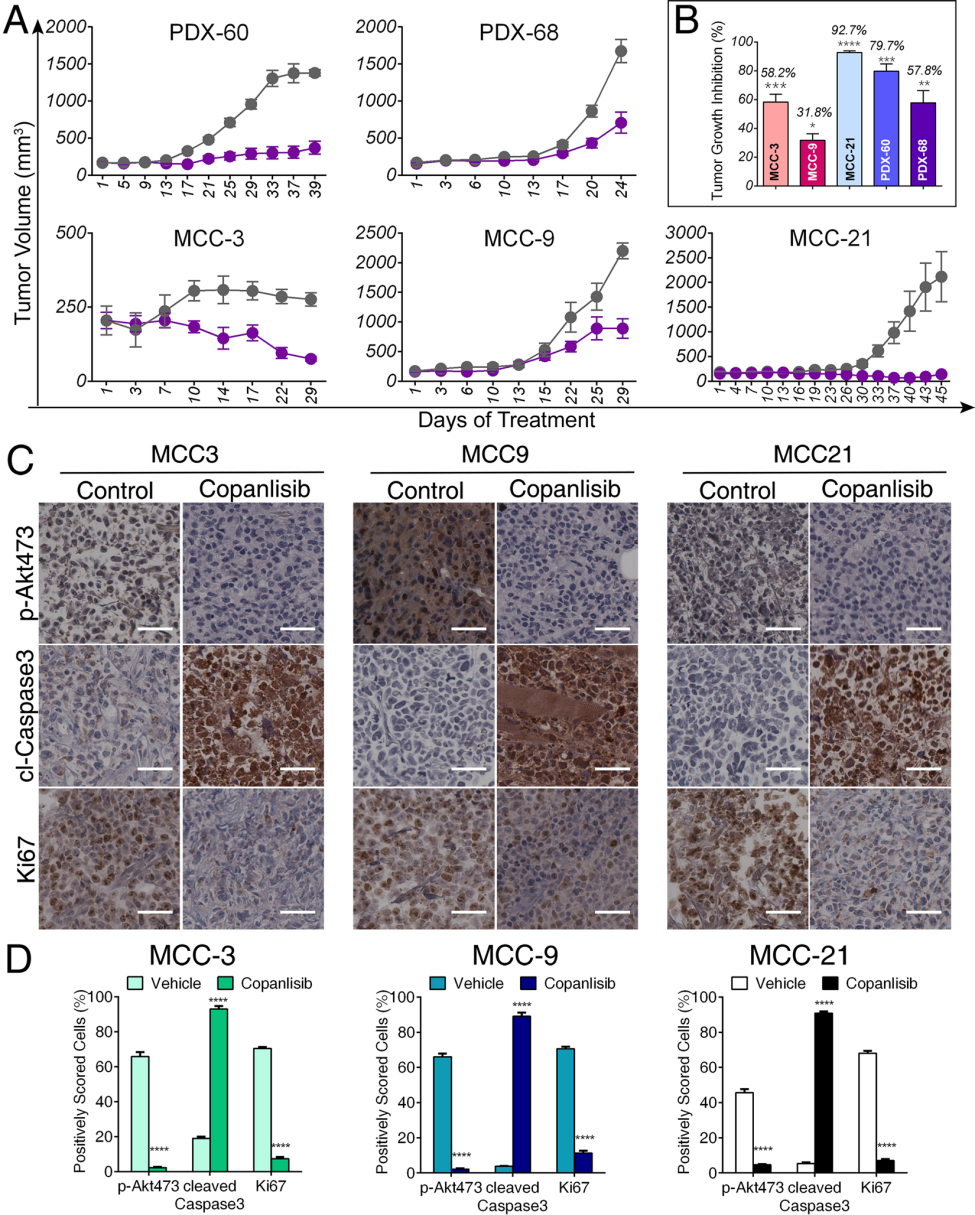
Copanlisib treatment attenuated tumor growth of MCC xenografts in mice. (**A**) NSG mice bearing MCC-3, MCC-9 or MCC-21 cell line-derived xenograft (CDX) tumors or patient-derived xenograft (PDX) tumors were treated with vehicle or copanlisib by intraperitoneal (i.p.) injection every other day for up to 6 weeks at doses and dosing schedules described in Materials and Methods. Tumor volume (TV) was measured every other day and reported as mean volume ± SD. (**B**) Tumor growth inhibition was calculated as (TGI) = [1-(TV_copanlisib_/mean TV_vehicle_)] × 100. (**C**) Immunohistochemical analysis of p-Akt473, Ki67 and cleaved caspase-3 was performed on MCC-3, MCC-9 or MCC-21 xenograft tumors treated with vehicle or copanlisib. Scale bars = 50μm. (**D**) Quantitative cell image analysis was carried out on tissue samples viewed at 400x magnification. Percentage of positive (brown nuclear/cytoplasmic staining) cells were scored. Vehicle-treated xenograft tumors served as negative controls. n = 5–10 * *p* < 0.05, **p < 0.01, ***p < 0.001, ****p < 0.0001 compared with vehicle-treated xenograft tumors by paired *Student t-test*.

Lastly, we investigated the *in vivo* anti-tumor efficacy of copanlisib using MCC cell line-derived xenograft (CDX) mouse models as described in our previous publications^49,59,60^. Matrigel was prepared with 2 × 10^7^ cells of MCC-3, MCC-9, and MCC-21, respectively, and inoculated subcutaneously into the rear flanks of immunodeficient NOD^scid^ gamma (NSG) mice. As described in Materials and Methods, we successfully established, for the first time, two MCC PDX models (PDX-60 and PDX-68). MCC cell lines and PDX tumors exhibited MCC histological features and classical MCC markers (Supplementary Figs. 1 and 2). When xenograft tumor growth approached ~100 mm^3^ in volume, mice began receiving 14 mg/kg of copanlisib or vehicle, administered by intraperitoneal injection every other day for up to 6 weeks. Copanlisib treatment had no obvious signs of toxicity as monitored by body weight, food and water intake, and activity (data not shown). As shown in Fig. 5A,B, copanlisib significantly attenuated *in vivo* growth of all three MCC CDX tumors and two PDX tumors. Of note, although the drug displayed more potent anti-tumor effects *in vitro* on MCC-3 and MCC-9 than MCC-21 (Fig. 2), copanlisib repressed MCC-21 tumor growth more markedly *in vivo*. The explanations and mechanisms for the differential effects of copanlisib on MCC *in vivo* and *in vitro* are unclear and warrants further study. Next, we performed immunohistochemistry staining of AKT phosphorylation, cleaved caspase-3, and Ki67, as indexes of PI3K activation, apoptosis, and cell proliferation, respectively, in paraffin-embedded xenograft tumor sections (Fig. 5C,D). Copanlisib treatment in mice led to significant inhibition of PI3K activity, induction of tumor cell apoptosis, and decrease in MCC cell proliferation *in vivo*. These data provide compelling evidence that dual inhibition of PI3K-α and -δ isoforms by copanlisib abrogates MCC tumor growth by inducing tumor cell apoptosis and inhibiting MCC cell proliferation.

## Discussion

Despite great advances in our understanding of MCC biology and therapy in recent years, the cellular and molecular mechanisms governing MCC tumorigenesis and metastasis remain largely unknown. Currently, no FDA-approved molecularly targeted therapy exists. Though immunotherapies targeting the PD1/PD-L1 immune checkpoint pathway have been FDA-approved for treatment of advanced MCC^11,12^, a significant proportion of MCC patients are either resistant to immune checkpoint blockade or unsuitable for immunotherapy due to autoimmune or immunosuppressed conditions^1^. There is an imperative need to identify and test novel targeted therapies, which can boost anticancer immunity in addition to their direct cell-autonomous effects on tumor cells, and can be used as alternative treatments for MCC patients who are not suitable for immunotherapy.

The molecules along the signaling network of PI3K/ATK/mTOR pathway regulate most cellular processes involved in cancer development, including cell cycle progression, survival, metabolism, motility and immunity^30^. Hyperactivation of this pathway is commonly detected in many types of cancers, including MCC^4,27,28,30,41^, and oncogenic mutations in *PIK3CA* gene have been detected in 4–10% of MCC^26,27^. Importantly, our group and others have shown promising anti-tumor effects on MCC by inhibition of PI3K and mTOR^26,27,47,49,58,59^.

Recently we first reported PI3K-δ expression in human MCC cells and the first successful clinical application of PI3K-δ inhibitor in a Stage IV MCC patient with *PIK3CA* mutation^47^. Consistent with the findings reported by Chteinberg *et al*.^48^, we have found that PI3K-δ isoform can be detected in 92% of archival MCC samples. However, we detected PI3K-δ expression in only 3 out of 4 MCC cell lines, including two MCPyV-negative cell lines (MCC-3 and MCC-9) and one MCPyV-positive cell line (MCC-21). Interestingly, we found only minimal PI3K-δ expression in MKL-1 cell line, which is inconsistent with the report by Chteinberg *et al*.

Although PI3K-α was the second most expressed isoform in our MCC cell lines, it was detected in 70% of archival MCC samples. In light of recent clinical success of copanlisib (PI3K-α/δ inhibitor) in treating breast cancer and other solid tumors, we examined antitumor efficacy of a panel of single and dual isoform-specific PI3K inhibitors including idelalisib (PI3K-δ), copanlisib (PI3K-α/δ), duvelisib (PI3K-γ/δ), alpelisib (PI3K-α), and AZD8186 (PI3K-β/δ). In two MCC cell lines (MCC-3 and MCC-9), we found that single isoform inhibition of PI3K-δ inhibitor (idelalisib) suppressed MCC cell growth more potently than PI3K-α inhibitor alone (alpelisib), but dual inhibition of PI3K-δ together with PI3K-α (copanlisib), PI3K-β (AZD8186), or PI3K-γ (duvelisib) suppressed MCC cell survival and proliferation more potently than PI3K-δ inhibition alone (idelalisib). A third MCC cell line, MCC-21, responded well only to alpelisib (PI3K-α) and copanlisib (PI3K-α/δ), suggesting that this cell line relies mainly on PI3K-α for PI3K activities despite its relatively higher mRNA expression of PI3K-δ.

We have previously demonstrated that idelalisib was able to resolve liver metastases in a patient with stage IV MCC^47^. However, we chose copanlisib for further *in vitro* and *in vivo* studies because, in addition to its relatively low GI_50_ value among the inhibitors tested, copanlisib has been recently approved by the FDA for treatment of breast cancer (solid tumor) with an acceptable side-effect profile, and we believe that it has greater translational potential^38^.

In contrast to our findings, Chteinberg *et al*. report that alpelisib (PI3K-α) more potently suppressed *in vitro* cell proliferation than idelalisib (PI3K-δ)^48^ in the panel of MCC cell lines tested in their laboratory. However, their reported half maximal inhibitory doses (IC_50_) of idelalisib, ranging from 29.6 µM to 81.9 µM, were well beyond the highest drug concentrations used in our dose response studies (10 µM). Moreover, Chteinberg *et al*. included two MCPyV-negative MCC cell lines (“MCC13” and “MCC26”), which have been characterized as atypical MCC cell lines^61^. Though the exact explanations for discrepancies between our two studies are unknown, differences in experiment design, cell culture conditions, and biochemical assays may contribute to the variance in our observations.

Although PI3K-α and -β expression was detected, MKL-1 cells were resistant to all five PI3K inhibitors tested. This may be due to the lack of PI3K/AKT/mTOR pathway activation in MKL-1 cell line, as we previously reported^49^, and suggests that this pathway plays a minimal role in MKL-1 tumorigenesis. Mechanistically, results from a series of cellular and biochemical experiments demonstrate that copanlisib inhibits PI3K/AKT/mTOR pathway activities and represses MCC cell proliferation and survival more potently than idelalisib in MCC-3, −9, and −21. Additionally, we found that copanlisib markedly suppresses growth and tumorigenesis of these three MCC cell lines *in vitro* as assessed by tumor cell colony formation assay, and *in vivo* as examined in three MCC CDX. These *in vivo* drug efficacy studies were further confirmed in two PDX mouse models of MCC; to our knowledge, this is the first reported preclinical drug study using MCC PDX models.

In the past few years, cancer immunotherapies targeting T-cell immune checkpoint receptors PD-1/PD-L1 have demonstrated great clinical benefits to MCC patients^11–13,17,18^. Nevertheless, 50% of MCC patients still succumb to their diseases despite immunotherapy, underscoring the need for new therapeutic strategies for those patients as well as those who are not suitable for immunotherapy due to immunosuppressed conditions and/or autoimmune diseases. These alternative therapies may also augment efficacy of immunotherapies and significantly improve clinical benefits when utilized in combination with different types of immune-targeting drugs^19–21^. A large-scale survey of cancer genomic and therapeutic databases has identified five candidate genes, namely *PIK3CA, BRAF, NF1, NRAS*, and *PTEN*, the targeting of which could be suitable for combination therapy with immunotherapy^62^. Moreover, inhibition of PI3K/AKT/mTOR pathway in other cancers has been shown not only to directly target cancer cells but also modulate tumor microenvironment and tumor-infiltrated immune cells^63–68^. Similar to other cancers, PI3K/AKT/mTOR pathway is hyperactive in MCC and inhibition of this pathway has demonstrated significant anti-MCC effects *in vitro* and *in vivo* as reported in this study and by others^26,27,47,49,58,59^.

In summary, we have confirmed abundant PI3K-δ expression in MCC and also demonstrated that PI3K-α is commonly expressed across MCC cell lines and archival MCC tumors. Furthermore, we have shown that inhibition of PI3K/AKT/mTOR pathway by copanlisib (PI3K-α/δ) suppresses MCC cell proliferation and survival more potently than other PI3K inhibitors with single/dual isoform specificities. Copanlisib attenuates MCC cell-line derived xenograft tumor growth by inhibiting MCC proliferation and stimulating apoptosis, and this therapeutic efficacy was further evaluated and confirmed in MCC patient-derived tumor xenograft models. Thus, this study provides compelling evidence for the application of copanlisib as monotherapy and/or potentially in combinatorial therapies for a subset of advanced MCCs, as well as other solid tumors with PI3K activation for which standard therapies are insufficient.

## Materials and Methods

### Compounds and reagents

Copanlisib, idelalisib (CAL101), AZD8186, alpelisib (BYL719) and duvelisib (IPI145) were purchased from Selleck Chemicals (Houston, TX). Inhibitors were prepared in sterile DMSO (final concentration <0.1%) and stored at −80 °C in small aliquots. Primary antibodies to Akt, mTOR, 4E-BP1, S6K, p-Akt-Ser473, p-Akt-Thr308, p-mTOR, p-PRAS40, p-4E-BP1, p-S6, p-GSK, cleaved caspase-3, cleaved-PARP, and PI3K-α were purchased from Cell Signaling Technology (Danvers, MA). Antibodies to PI3K-δ, Ki67, and α-Tubulin were obtained from Santa Cruz Biotechnology (Dallas, TX), Abcam (Cambridge, MA), and Sigma-Aldrich (St. Louis, MO), respectively. RPMI-1640 and Dulbecco’s Modified Eagle’s Medium (DMEM) were purchased from American Type Culture Collection (ATCC, Manassas, VA). Fetal bovine serum and tissue culture supplements were obtained from Atlanta Biologicals (Flowery Branch, GA) and Life Technologies (Houston, TX), respectively. Additional reagents include RIPA buffer (Sigma-Aldrich) and enhanced chemiluminescence (ECL) detection reagent (Millipore).

### Generation of MCC cell line-derived and patient-derived xenograft models in mice

MCC cell line-derived xenograft (CDX) mouse models were generated using six-week-old female immunodeficient NSG mice (Jackson Laboratory, strain #005557). In brief, 2 × 10^7^ MCC cells were suspended in Matrigel (BD Biosciences; catalog # 354248) and subcutaneously inoculated on right rear flanks. Palpable tumor growth appeared within 3 to 5 days of inoculation, and treatment per protocol began when tumors reached approximately 100 mm^3^ volume. To generate MCC patient-derived xenograft (PDX) mouse models, we obtained excess surgical tissue from consenting MCC patients at the University of Arkansas for Medical Sciences (UAMS) in accordance with the Declaration of Helsinki and relevant institutional guidelines for human studies, under study protocols approved by the Institutional Review Board (IRB). Animal protocols were approved by the Institutional Animal Care and Use Committee (IACUC) at UAMS, in accordance with laboratory animal care and use guidelines set by the Association for Assessment and Accreditation of Laboratory Animal Care (AAALAC) International. Briefly, excess fresh MCC tissues not needed for clinical diagnosis were processed and sectioned into 2- to 4-mm^3^ pieces. Non-necrotic pieces were subcutaneously implanted into the rear flanks of immunodeficient NSG mice. Per standard parlance^69,70^, this initial engraftment of human tumor tissue was termed as “F_1_” generation; successful engraftments were subsequently allowed to grow until approaching tumor endpoint (~1500mm^3^ volume), harvested, processed, biobanked and/or passaged into further immunodeficient NSG mouse cohorts. Each successive mouse-to-mouse passage was numbered consecutively as F_2_ generation and so forth. RNA, gDNA, and whole tissue samples were obtained from tumors in each generational cohort, characterized by RT-PCR and immunohistochemistry, and compared to originating tumors to validate each MCC PDX lineage. In this study we utilized F_5_ generation of our PDX-60 lineage and F_6_ generation of PDX-68 lineage, which exhibit classical MCC morphology and express classic MCC markers (see Supplementary Fig. 2), to expand tumor-bearing mouse cohorts for copanlisib preclinical drug studies. Tumor-bearing mice were randomly divided into control and treatment groups (n = 5–10 for each condition) receiving copanlisib treatment. Copanlisib was formulated in PEG400/acidified water solution with pH ~4.5 and administered at 14 mg/kg via intraperitoneal injection every other day. Control mice received vehicle only. Mice were monitored daily and tumors were measured using digital calipers. Tumor volume (TV) was calculated as *L* x *W*^2^/2, where length (L) is the longer dimension and width (W) is the shorter dimension. The therapeutic efficacy of copanlisib on tumor growth in each CDX and PDX was defined by tumor growth inhibition, calculated as (TGI) = [1−(TV_copanlisib_/mean TV_vehicle_)] × 100.

### Immunohistochemistry

Dissected MCC xenograft tumors and MCC patient tumor samples (collected under protocols approved by the UAMS IRB in accordance with relevant guidelines) were fixed overnight in 10% neutral buffered formalin and paraffin-embedded by routine histology procedure. Five micrometer tissue section slides were prepared, processed for antigen retrieval, and stained as described before^49,58,59^. Samples were incubated with specific primary antibodies for p-Akt-Ser473 (1:50), cleaved caspase-3 (1:100) and Ki67 (1:2000) at 4 °C overnight. Samples were then incubated with goat anti-rabbit-secondary antibody for one hour at room temperature, followed by development with horseradish peroxidase detection system. Slides were viewed under an Olympus BX51 Research System Microscope and images were captured using a high-resolution interline CCD camera at 400x magnification. Positively stained cells were quantified in 5 randomly chosen fields per slide, and three slides per group were used for each stain. Data are presented as the proportion of positively stained cells over the total number of cells.

### Cell culture

MCC cell lines (MCC-3, MCC-9, and MCC-21) were established in our laboratory under study protocols approved by the UAMS IRB, in accordance with the Declaration of Helsinki and relevant regulations. MKL-1, a well characterized MCPyV-positive cell line, was gifted by Dr. Becker (Department of Dermatology, University Hospital Essen, Essen, Germany). MCC cells grow in clusters in suspension, and are maintained in RPMI-1640 medium supplemented with 10% FBS and penicillin-streptomycin (100units/ml) and L-glutamine (4 mM) at 37 °C in a humidified atmosphere with 5% CO_2_. Cells were fed fresh complete media every other day and split 1:2 weekly to maintain logarithmic growth. MCC cell lines were authenticated via STR-profiling (Genetica, Burlington, NC), comparing each MCC cell line against respective primary MCC tumor^49,58,59^; see Supplementary Data.

### Cell proliferation and viability assay

Cell proliferation and viability were measured by Cell Counting Kit-8 (Sigma-Aldrich) per manufacturer’s protocol. In brief, cells were plated at 1 × 10^4^ cells per well in 96-well plates, allowed to recover for 4 hours, then exposed to serial concentrations of idelalisib, alpelisib, copanlisib, AZD8186, and duvelisib for 72 hours. CCK-8 (10% of well volume) was added to each well and incubated for 4 hours at 37 °C before recording optical density (OD) at 450 nm using a spectrophotometer. Maximal cell proliferation was defined by the average OD of the control condition minus background. Half-maximal growth inhibitory dose (GI_50_) was calculated by plotting dose-response curve and identifying the concentration at which 50% of maximal cell proliferation was suppressed.

### Methylcellulose colony-forming assay

To evaluate colony formation, MCC cells were cultured in complete methylcellulose medium (MethoCult GF M3434, Stem Cell Technologies, Vancouver, Canada) according to manufacturer’s protocol. Briefly, MCC cells (25,000 cells) were resuspended in complete methylcellulose with 50 nM copanlisib or vehicle, plated in 35 mm plates, and maintained in 37 °C incubator. Clusters consisting of ≥40 cells were counted, scored, and imaged on day 14 post-seeding.

### Cell cycle analysis by flow cytometry

Cell cycle distribution in MCC cell populations was detected by BD Pharmingen BrdU Flow Kits (BD Biosciences; San Jose, CA). MCC cells were seeded at a cell density of 2 × 10^5^ per well in 6-well plates and treated with idelalisib, alpelisib, copanlisib, AZD8186 and duvelisib for 24 hours as described before^59^. BrdU incorporation was detected using FITC-conjugated anti-BrdU antibody followed by 7-AAD staining per manufacturer’s protocol. Cell cycle detection was performed via FACSAria flow cytometer and analyzed by FlowJo software (version 10.4.2) and cell cycle distribution was reported as the percentage of cells in G0/G1, S, and G2/M populations.

### Determination of apoptosis by flow cytometry

Apoptotic cells in each control and treatment group were detected by Annexin V-FITC apoptosis-detection kit (BD Biosciences; San Jose, CA). Briefly, MCC cells were plated in 6 well plates (2 × 10^5^ per well) and treated with idelalisib, copanlisib, AZD8186 or duvelisib for 24 hours at indicated concentrations. At the end of incubation, cells were collected and stained with Annexin-V-FITC/Propidium Iodide (PI) followed by FACSAria (BD Biosciences) analysis within an hour of staining. Cell death was scored by the following criteria, set by appropriate gating: (a) early apoptotic cells (PI negative, FITC Annevin-V positive), (b) late apoptotic or dead cells (doubly positive for both FITC Annevin-V and PI), and (c) live cells (doubly negative for Annexin-V and PI). Statistical analysis was performed using FlowJo software (version 10.4.2).

### RNA extraction and gene expression analysis

Total RNA was isolated from MCC cells via RNeasy Kit (Qiagen) per manufacturer’s instructions. Complementary DNA was generated from MCC mRNA using High-Capacity cDNA Reverse Transcription Kit (Life Technologies). Quantitative real-time-PCR (qRT-PCR) was performed with a StepOne Plus Real-Time PCR System (Applied Biosystems) as described previously using specific TaqMan Gene Expression Assay primers purchased from Applied Biosystems: PI3Kα, PI3Kβ, PI3Kγ and PI3Kδ and MRPS2 (mitochondrial ribosomal protein S2). Triplicate runs of each sample were normalized to MRPS2 mRNA to determine relative expression.

### Western blot

MCC cells were harvested and processed for Western blot analysis as described previously^49,58,59^. Xenograft tumor tissues harvested from mice were homogenized in 2% SDS lysis buffer and processed as described previously^49^. Briefly, whole cell protein lysates (10–30 µg per lane) were resolved by 8% or 12% SDS-PAGE gel electrophoresis and transferred onto PVDF membranes by a semidry blotting system (Bio-Rad, Hercules, CA). Membranes were blocked in 5% fat-free milk/Tris–buffered saline for 1 hour at RT and incubated with specific primary antibodies at 4 °C overnight, followed by one hour RT incubation with secondary antibodies conjugated with horseradish peroxidase. Visualization of immunoreactive proteins was achieved using ECL detection reagent per manufacturer’s instruction. Alpha-tubulin was used as a loading control and all immunoblotting data represent contiguous lanes.

### Statistical analysis

All measurements were made in triplicate, and all values are represented as mean ± SD. Statistical analyses were performed with *Student t test* or one-way analysis of variance (ANOVA) using GraphPad prism software (v6.0; San Diego, CA). *P* value < 0.05 was considered statistically significant.

## Supplementary information

Supplemental information.
