# Supplementary material for: Inhibition of PI3K by copanlisib exerts potent antitumor effects on Merkel cell carcinoma cell lines and mouse xenografts
Source: Sci Rep. 2020 Jun 1;10:8867. doi: 10.1038/s41598-020-65637-2 (PMC7264292; doi:10.1038/s41598-020-65637-2)
Supplement: Supplementary file 1 — Supplemental information. [file 41598_2020_65637_MOESM1_ESM.pdf]

Supplementary Data

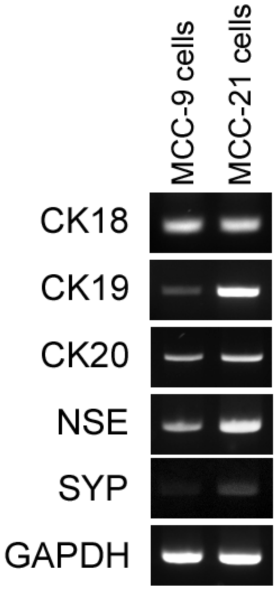

Supplementary Figure 1. Expression of MCC markers in MCC-9 and MCC-21 cell lines Uncropped gels below.

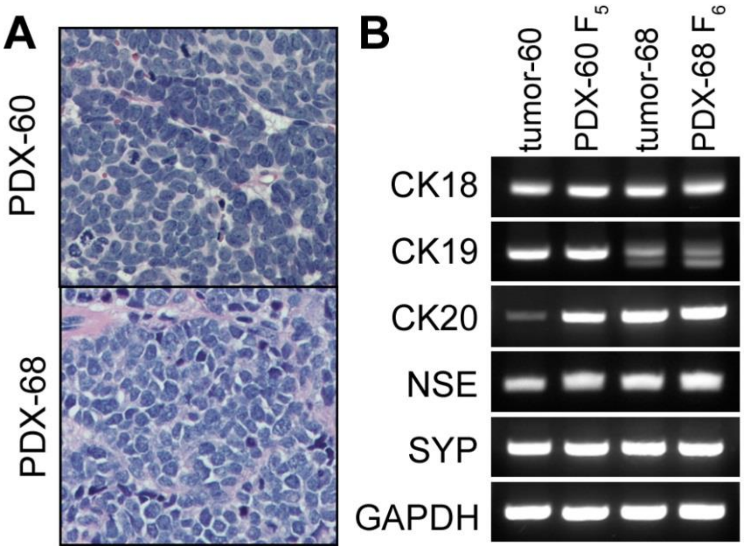

Supplementary Figure 2. Characterization of two MCC PDX lineages. (A) Hematoxylin and eosin staining of original tumor and PDX tumors, illustrating large nuclei with scant cytoplasm. (B) Expression of MCC markers by RT-PCR analysis. Uncropped gels below.

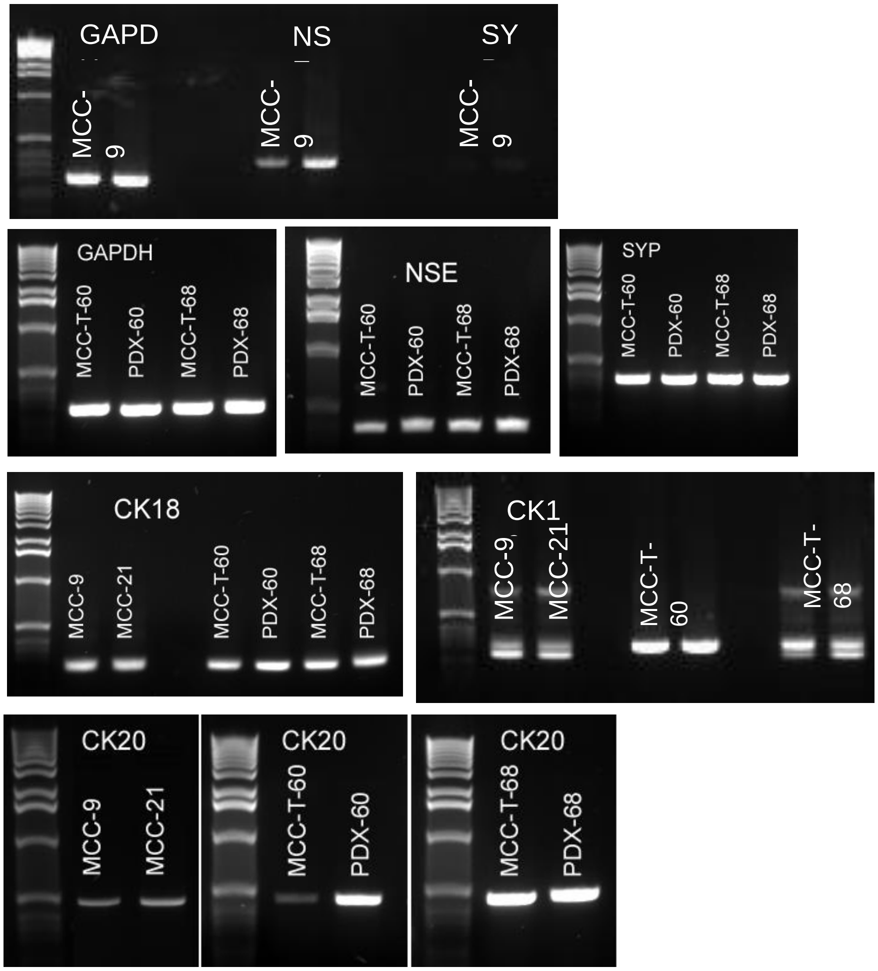

Supplementary Figure 3. Original immunoblottings from Figure 4.

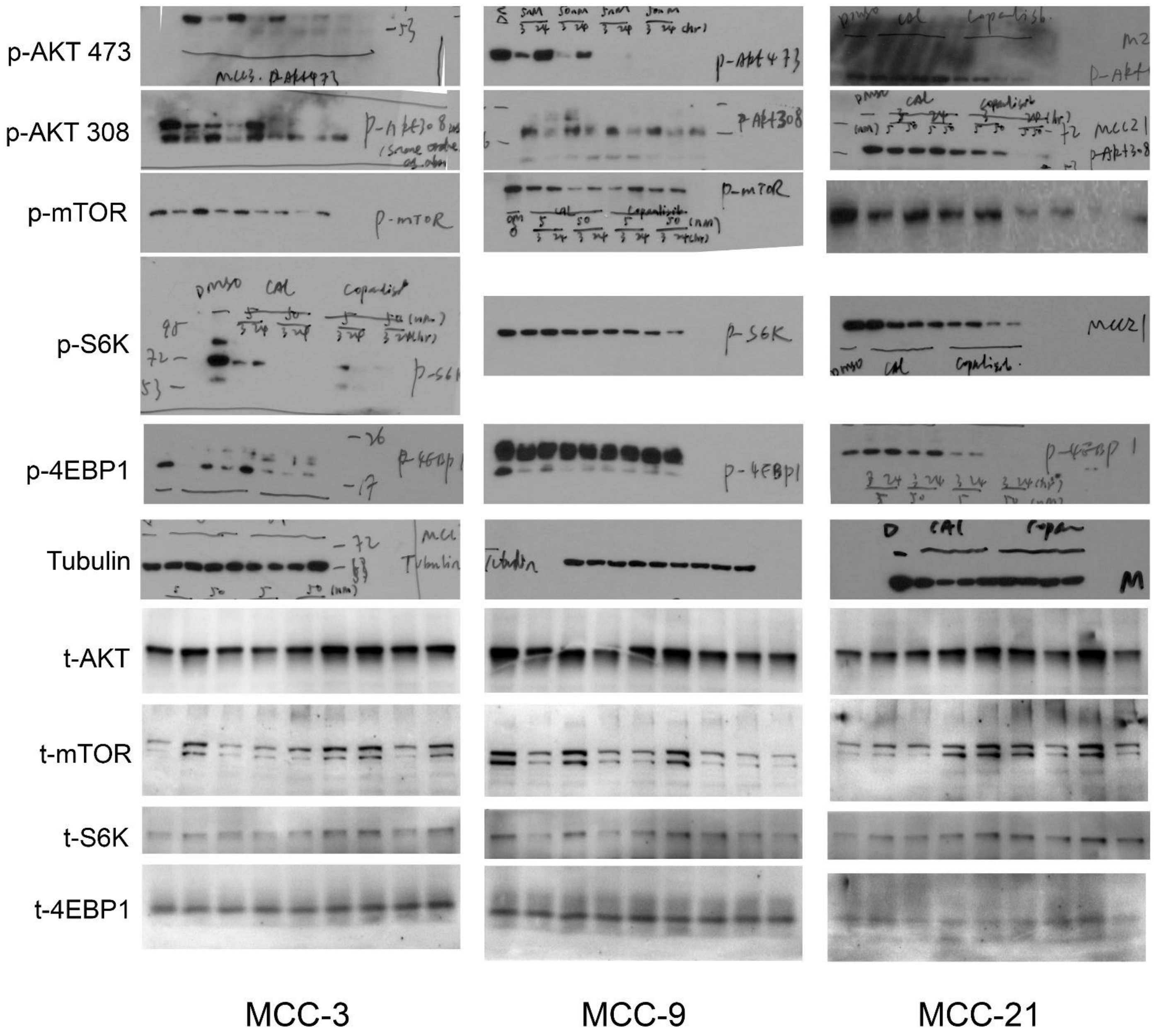

Supplementary Figure 4. Copanlisib fails to induce apoptosis in MKL-1 cells. MKL-1 cells were treated with copanlisib (1µM) for 24 hrs; DMSO-treated cells served as controls. Cells were stained by Annexin-V and PI (propidium iodide) and analyzed by flow cytometry; percentages of Annexin V^+, PI^- (early apoptotic) and Annexin-V^+, PI^+ (late apoptotic) cells were calculated in each group. Bar graphs (±SD) represent all dead cells including Annexin V^+, PI^- cells and Annexin-V^+, PI^+ cells.

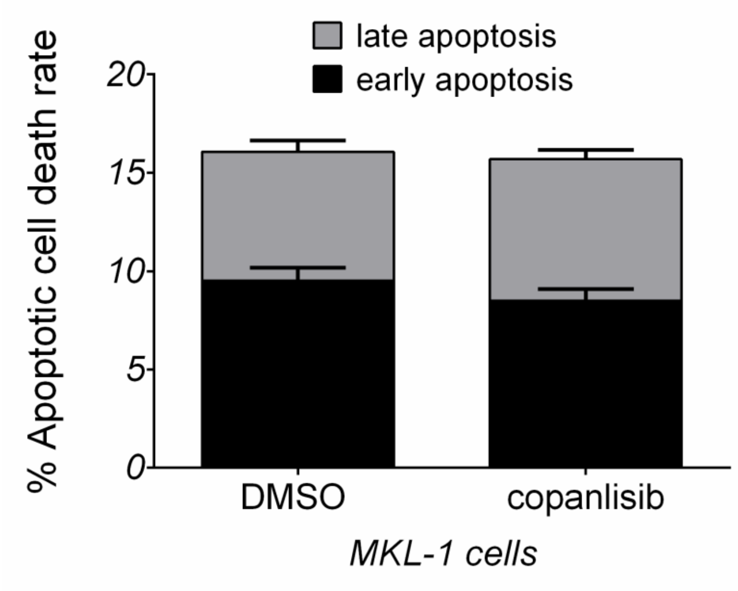

Supplementary Table 1. Authentication of MCC-9 and MCC-21 cell lines. STR-profiling was performed by Genetica (Burlington, NC).

DNA Analysis by STR-profiling

MCC-9	D3S1358	D7S820	vWA	FGA	D8S1179	D21S11	D18S51	D5S818	D13S317
MCC-9 cell line	14	10, 11	18, 20	20, 24	10, 14	28, 32.2	13, 15	11, 12	10, 12
MCC-9 originating tumor	14	10, 11	18, 20	20, 24	10, 14	28, 32.2	13, 15	11, 12	10, 12

	D16S539	TH01	TPOX	CSF1PO	AMEL	Penta D	Penta E	Mouse
MCC-9 cell line	12	7	8, 9	10	X, Y	9, 14	7, 10	NA*
MCC-9 originating tumor	12	7	8, 9	10	X, Y	9, 14	7, 10	NA

MCC-21	D3S1358	D7S820	vWA	FGA	D8S1179	D21S11	D18S51	D5S818	D13S317
MCC-21 cell line	17	8, 10	15, 16	21, 23	9	30, 32.2	13, 15	12	11
MCC-21 originating tumor	17	8, 10	15, 16	21, 23	9	30, 32.2	13, 15	12	11

	D16S539	TH01	TPOX	CSF1PO	AMEL	Penta D	Penta E	Mouse
MCC-21 cell line	11, 12	6, 9.3	9, 11	11, 12	X	10, 13	7, 14	NA*
MCC-21 originating tumor	11, 12	6, 9.3	9, 11	11, 12	X	10, 13	7, 14	NA
